# What Can Restructuring Laws Do? Geopolitical Shocks, the New German Restructuring Regime, and the Limits of Restructuring Laws

**DOI:** 10.1007/s40804-023-00271-9

**Published:** 2023-03-16

**Authors:** Horst Eidenmüller

**Affiliations:** 1 University of Oxford, Oxford, UK; 2 ECGI Research Associate, European Corporate Governance Institute, Brussels, Belgium

**Keywords:** Insolvency, Bankruptcy, Restructuring, Restructuring laws, Covid-19, Energy crisis, Ad hoc bailout

## Abstract

In this article, I discuss the possibilities and limitations of restructuring laws against the background of geopolitical shocks such as the Covid-19 pandemic and the current energy crisis. I make two claims, one narrow and focused on German bankruptcy law, and one broad with a cross-jurisdictional reach. My narrow claim relates to ‘StaRUG’, the new German restructuring regime. I argue that this law is a superfluous and flawed instrument. It should be repealed. My second claim is much broader. I argue that bankruptcy laws, including restructuring laws, are generally ill-suited to deal with the economic consequences of geopolitical or macroeconomic shocks as a ‘first line of defence’. Bankruptcy laws are not designed to provide the structural assistance at scale which the businesses affected by these shocks need. At the same time, massive state aid for distressed businesses in times of crisis and, in particular, ad hoc bailouts of large critical firms are also problematic. I propose that firms’ resilience against geopolitical or macroeconomic shocks should be strengthened, and that best practices (‘Principles’) for bailouts should be developed.

## Introduction

Corporate bankruptcy law is a tool to resolve the financial distress of corporations. Unviable corporations are liquidated and the proceeds distributed to the creditors. Viable corporations are restructured and put on a new financial footing. Creditors receive claims against the restructured entity and/or cash. Following the iconic model of Chapter 11 of the US Bankruptcy Code,^1^ many jurisdictions worldwide have strengthened or introduced such restructuring options in their bankruptcy laws. In the European Union (EU), for example, a ‘Restructuring Directive’ was adopted in 2019 which seeks to harmonise Member States’ pre-insolvency restructuring regimes.^2^ Germany implemented this Restructuring Directive with a new restructuring law, the *Unternehmensstabilisierungs- und -restrukturierungsgesetz* (‘StaRUG’), which entered into force on 1 January 2021.^3^

Bankruptcy laws, including restructuring laws, are usually not designed to process the financial failure of masses of similarly situated firms. The typical scenario is the financial distress of an individual firm which may or may not have a viable business model. Of course, bankruptcy laws are also capable of managing the simultaneous influx of a greater number of distressed firms if, for example, a certain business sector is disrupted by innovation or particularly affected by an economic downswing. But geopolitical and macroeconomic shocks and the associated economic fallout in the sense of millions of struggling firms are a different matter. The run to the bankruptcy court might bring the machinery of bankruptcy justice to a standstill.

Since the beginning of 2020, the world has seen two such shocks. The first came with the Covid-19 pandemic. Governments worldwide imposed lockdowns. Firms could not trade and lost revenues on an unprecedented scale. Governments extended massive packages of relief to businesses, often in the form of loans. Keeping businesses out of bankruptcy appeared to be the overriding goal—at enormous costs to the taxpayer. Again, Germany is a case in point, as ably demonstrated by Wolfram Prusko and David Ehmke in their contribution to this volume.^4^ The second shock has come with the war in Ukraine. Energy costs have soared, supply chains have been disrupted. Once more, governments have stepped in with energy cost subsidies and other forms of financial assistance for businesses, seeking to prevent mass bankruptcy filings.

In this article, I investigate the merits of these anti-bankruptcy policies. I make two claims, one narrow and focused on German bankruptcy law, and one broad with a cross-jurisdictional reach. My narrow claim relates to the new German restructuring law mentioned above, i.e., the StaRUG. I will argue that this law is a superfluous and flawed instrument. It should be repealed. The case against the StaRUG does not rest on arguments about shock events. Rather, the StaRUG is just a particularly unuseful restructuring tool. My second claim is much broader. I will argue that bankruptcy laws, including restructuring laws, are generally ill-suited to deal with the economic consequences of geopolitical or macroeconomic shocks. Bankruptcy laws are not designed to provide the structural assistance at scale which the businesses affected by these events need. Hence, the post-Covid anti-bankruptcy policies of many states, including Germany, are justified in principle. At the same time, massive state aid for distressed businesses in times of crisis and, in particular, ad hoc bailouts of large firms are also problematic. I propose that firms’ resilience against geopolitical or macroeconomic shocks should be strengthened, and that best practices (‘Principles’) for bailouts should be developed.

## The New German Restructuring Regime

Since 1 January 1999, the central insolvency statute in Germany has been the *Insolvenzordnung* (Insolvency Code, ‘InsO’). It is a multi-purpose tool: insolvent businesses can be liquidated or sold as a going concern. They can also be restructured. Sections 217–269 InsO contain the so-called ‘Insolvency Plan Procedure’. Its main use is to restructure a distressed business based on an Insolvency Plan. The procedure adapts, to a large degree, Chapter 11 of the US Bankruptcy Code. Some of the Insolvency Plan Procedure’s provisions such as, for example, the cross-class cram down (Section 245 InsO), are almost literal transplants of the corresponding Chapter 11 provision (in this case: 11 USC § 1129(b)).^5^ Crucially, the debtor can also access an Insolvency Plan Procedure and, indeed, any insolvency procedure under the InsO, pre-insolvency if insolvency is ‘imminent’ (Section 18(1) InsO). Section 18(2) InsO stipulates that ‘[a] debtor is deemed to be faced with imminent insolvency if it is likely that the debtor will be unable to meet existing obligations to pay on the date of their maturity. The forecasting period is generally to be 24 months.’ Hence, the Insolvency Plan Procedure can be used as a pre-insolvency restructuring tool.

Since 1999, the Insolvency Plan Procedure has become an established feature of the German bankruptcy system. Unfortunately, the German Federal Statistical Office only records the total number of business insolvencies per year but not the number of Insolvency Plan Procedures. In 2021, 13,993 businesses filed for insolvency proceedings.^6^ An Insolvency Plan Procedure is not necessarily a debtor-in-possession (DIP) proceeding. However, in practice the correlation is strong. Since 1999, the number of DIP proceedings has fluctuated between 132 (minimum, in 2000) and 420 (maximum, in 2013); in 2021, the number was 210.^7^ It therefore appears that an Insolvency Plan Procedure is probably used in only 1–3% of all business insolvencies. This is certainly not a high figure. But the Insolvency Plan Procedure has been and *is* being used for business restructurings on a continuing basis since 1999, especially in high-profile cases. A recent example is the restructuring of Sunline AG, a listed photovoltaic company.^8^

As mentioned in the Introduction, the European lawmaker adopted a Restructuring Directive in 2019. It seeks to harmonise Member States’ pre-insolvency restructuring regimes. Germany has been discussing the need for a special pre-insolvency restructuring regime for businesses for many years before the advent of the European Restructuring Directive. It was in the aftermath of the great financial and economic crisis, which started in 2007, that the issue first came up. The proponents of a dedicated pre-insolvency restructuring law argued that Germany needed a proceeding akin to the English Scheme of Arrangement to stay competitive in the international market for the best restructuring law product.^9^ Others argued that no such proceeding was necessary.^10^ The InsO had been introduced approximately 10 years earlier precisely to end the multiplicity of proceedings which existed before it came into force. As already mentioned, the InsO is a multi-purpose tool, and it can be accessed by the debtor pre-insolvency to restructure its distressed business. At the time, the opponents of a dedicated pre-insolvency restructuring proceeding won the argument. No such proceeding was adopted, and the matter appeared to be settled.

Foreseeably, the debate was revitalised with the European Restructuring Directive in 2019, requiring EU Member States to comply with it by 17 July 2021 (Article 34(1) Restructuring Directive). The German lawmaker could have taken the position that no further action needed to be taken. An article-by-article analysis of the Restructuring Directive would have led one to conclude that the Insolvency Plan Procedure was fully compliant with the mandates of the Restructuring Directive. In particular, as discussed, the Insolvency Plan Procedure could be initiated pre-insolvency, and it could be run as a DIP proceeding without the appointment of an insolvency administrator. However, in the political realm and in the perception of the general public, the Insolvency Plan Procedure, as the Insolvency Code (InsO) more generally, was perceived by many as an *insolvency* proceeding alone and not as a pre-insolvency restructuring tool. And it is of course politically much easier to tell Brussels that one has adopted a new dedicated restructuring regime in transposition of the Restructuring Directive than to make the argument that, despite its name, the Insolvency Plan Procedure actually meets all the Directive’s requirements and that no further action is necessary.

However, what eventually pushed the *Unternehmensstabilisierungs- und -restrukturierungsgesetz* (‘StaRUG’) over the line politically in 2020/2021 was the Covid-19 pandemic. As discussed in detail in Wolfram Prusko’s and David Ehmke’s contribution, the German government adopted a hotchpotch of measures during the spring/summer of 2020 which sought to contain the economic fallout from the pandemic. A key element of the German strategy was to keep Covid-distressed businesses outside of bankruptcy proceedings by suspending filing duties and offering generous cash flow assistance.^11^ The scope of, and access to, an existing furlough scheme was broadened to maintain employment levels, and a temporary reduction of VAT from 19 to 16% was introduced to stimulate consumer demand. A new restructuring regime for those businesses which, despite the generous financial assistance offered by the state, faced existential financial distress, seemed to be the missing piece in a complex regulatory puzzle. Hence, the StaRUG made it onto the statute book.

The new German restructuring regime has not been a success. No official statistics of the number of StaRUG proceedings since 1 January 2021 are or will be available as these proceedings are confidential. Based on hand-collected data from a private insolvency data tracker, 22 firms initiated StaRUG proceedings in 2021.^12^ A leading expert on the StaRUG expects that the number of StaRUG proceedings in 2022 will be even lower.^13^

**Fig. 1:**
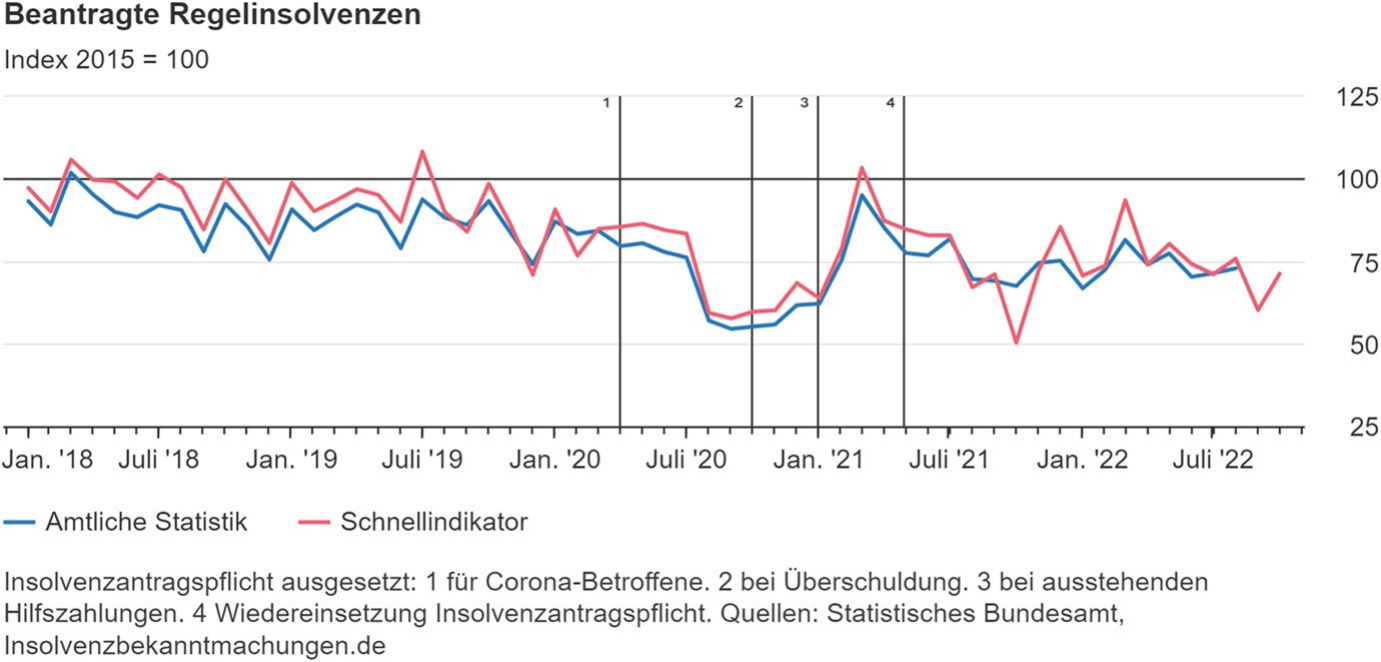
Regular insolvency proceedings in Germany over time (2018–2022). The blue line indicates official statistics, while the red line the results of a flash survey. The note reads: ‘Insolvency filing duty suspended: 1. for companies affected by corona, 2. in cases of over-indebtedness, 3. for companies with outstanding state support payments. 4. reinstatement of filing duty.' Source: German Federal Statistical Office, Press Release No. 475, dated 11 November 2022, https://www.destatis.de/DE/Presse/Pressemitteilungen/Aktuell/aktuelle-insolvenzen.html (last visited on 12 November 2022)

Figure 1 shows key insolvency data from Germany from 2018 to 2022 based on statistics from the German Federal Statistical Office. It depicts ‘regular insolvency proceedings’ over time against a baseline index of 100 defined by the number of such proceedings in 2015. ‘Regular insolvency proceedings’ comprise all insolvency proceedings except consumer bankruptcies. As can be seen from the chart, with the Covid support and anti-bankruptcy measures kicking in mid-2020, the number of ‘regular insolvency proceedings’ declined. Even after these measures came to an end, bankruptcy proceedings are currently still at only 75% of the figures for 2015.

As already discussed, the overwhelming majority of these proceedings are not restructuring proceedings. The share of Insolvency Plan Procedures is no more than 1–3% of the total number of business insolvencies. The share of StaRUG proceedings is even lower: in absolute terms, there were 210 DIP proceedings and approximately as many Insolvency Plan Proceedings in 2021 compared to 22 StaRUG proceedings. Hence, the share of StaRUG proceedings was no more than 0.1–0.3% of all business insolvencies in 2021—an almost negligible fraction. The figure for 2022 could be even lower.

What explains this almost negligible relevance of the new German restructuring regime? The reasons have nothing to do with the general anti-bankruptcy policy of the German government in its attempt to contain the economic fallout from Covid-19. To the contrary, the direction of this policy was to encourage early corporate restructurings, and the StaRUG was to be the legislative tool with which to achieve this.

The reasons for the failure of the StaRUG are to be found in the StaRUG itself. It is an unnecessary and flawed instrument and should be repealed. I have already argued that the StaRUG was unnecessary. The Insolvency Plan Procedure of the InsO can be accessed pre-insolvency to achieve a restructuring of a distressed business.

Compared to the Insolvency Plan Procedure, the StaRUG is an inferior debt restructuring instrument. It cannot be accessed earlier than the Insolvency Plan Procedure because it also requires ‘imminent insolvency’ within the meaning of Section 18(2) InsO for the debtor to be able to initiate a StaRUG proceeding (Section 29(1) StaRUG). The courts appear to take this entry requirement seriously.^14^ It is worth repeating this: the new German early restructuring regime cannot be initiated earlier than an insolvency proceeding.

This is so because the StaRUG is, in essence, not a light-touch and simple pre-insolvency restructuring proceeding, but unnecessarily complex, detailed and cumbersome. The new restructuring law is more than twice as long in terms of word count than the Insolvency Plan Procedure of the InsO. It contains all the provisions of a ‘structured bargaining procedure’ as found in the InsO, and more. Central to a StaRUG proceeding is a ‘restructuring plan’. The plan can modify creditors’ claims and security rights, and it can also involve debt-to-equity swaps (Section 2 StaRUG). Creditors’ claims are grouped in a restructuring plan, and StaRUG features a cross-class cram down mechanism as part of the plan approval process (Sections 25-29 StaRUG). There is a variety of new tools compared to the Insolvency Plan Procedure of the InsO, such as ‘stabilisation orders’ (Sections 49–59 StaRUG—these provisions replace the automatic stay applicable in an InsO proceeding), as well as provisions on a ‘restructuring official’ (Sections 73–83 StaRUG) and a ‘restructuring moderation’ (Sections 94–99 StaRUG).

In essence, the StaRUG proceeding is a blown-up version of the Insolvency Plan Procedure. It is not obvious whether and how the new detailed provisions bring improvements compared to the Insolvency Plan Procedure. However, it is obvious that these provisions make matters more complicated and cumbersome, increasing transaction costs. Germany now has two restructuring regimes: the classic Insolvency Plan Procedure, and an expanded Insolvency Plan Procedure, i.e., the StaRUG.

How could this have happened? What is the point in taking an existing restructuring tool, making it more complex, adding a couple of new features, and making *both* the original and the prettified replica available to market participants? Arguably, the likely consequence is that the attractivity of both instruments might suffer: the classic Insolvency Plan Procedure might come to be seen as old-fashioned and outdated, while the new StaRUG, untested and complicated as it is, might deter debtors and fail to attract a significant number of users in the first place—as the numbers for 2021 and 2022 appear to indicate.

I submit that what happened is an example of corrupted public choice and regulatory capture. What has been going on for decades in the background is a power struggle between two influential groups of insolvency/restructuring professionals: insolvency administrators on the one hand^15^ and restructuring professionals in law and accounting firms on the other.^16^

German insolvency proceedings have been dominated for a long time by insolvency administrators. Section 27 (1) InsO stipulates that the insolvency court appoints an insolvency administrator when resolving to open an insolvency proceeding. True, the insolvency court can also order that the proceeding be run as a DIP proceeding (Sections 27 (1), 270 InsO). However, German insolvency practice was, for a long time, characterised by a deep distrust of such proceedings. It was felt that allowing the debtor to run the insolvency proceeding would amount to putting the fox in charge of the henhouse.^17^ Hence, it was argued that court-appointed insolvency administrators are needed to make sure that the proceedings are run in an orderly fashion. Matters have changed over the last two decades, but DIP proceedings and the Insolvency Plan Procedure are still a rather marginal feature of the German bankruptcy landscape.

The restructuring professionals in law and accounting firms do not like this. In regular insolvency proceedings they do not sit in the driver’s seat, and they do not earn the largest fees. Instead, it is the insolvency administrators who run the show and make the most money. The insolvency administrator of the German Lehman subsidiary reputedly earned a fee in the order of 300 to 800 million euro, for example.^18^ By pushing for the adoption of the StaRUG,^19^ the restructuring professionals in law and accounting firms attempted to increase their share of the huge fees which can be earned in a corporate restructuring.

However, for this plan to work, the StaRUG proceedings needed to be DIP proceedings or be run with as light-touch court supervision as possible. The European Restructuring Directive allows this (Article 5). But the German insolvency administrators pushed back,^20^ as was to be expected. The end result is 11 long and detailed provisions on the appointment, functions, conduct and fees of a ‘restructuring official’ (Sections 73–83 StaRUG). These provisions are an overly complicated regulatory compromise between the insolvency administrators and the restructuring professionals in law and accounting firms. The insolvency administrators pushed for routine appointments of old-style insolvency administrators from their ranks. The restructuring professionals pushed for DIP proceedings and, as a second-best solution, for appointments of restructuring experts from their ranks. The outcome of the regulatory haggling is an overly complex compromise solution.

To sum up, for almost 25 years, Germany has had a functioning restructuring proceeding, the Insolvency Plan Procedure. It is certainly not hugely popular. But it is an established feature of the German bankruptcy landscape, it works, and, importantly, it can be accessed pre-insolvency. The European Restructuring Directive did not require the German lawmaker to introduce a new restructuring regime. The StaRUG is a superfluous instrument. But it is also a flawed instrument. It is overly complex and cumbersome. The StaRUG is the product of regulatory capture by the restructuring industry. It should be repealed.

## The Limits of Restructuring Laws

As discussed in Section 2, a key goal of the German government’s regulatory response to the Covid-19 pandemic was to keep Covid-distressed businesses outside of bankruptcy proceedings by suspending filing duties and offering generous cash flow assistance. Enormous amounts of assistance have also been earmarked to reduce the impact of the current energy crisis, following the war in Ukraine. On 21 October 2022, the German parliament approved the government’s 200 billion euro rescue package that aims to protect companies and households from the impact of soaring energy prices.^21^

Despite their scale, these measures were and are not sufficient to prevent the financial distress of all businesses. The overwhelming majority of these distressed businesses did or will end up in bankruptcy proceedings, including the Insolvency Plan Procedure or the new StaRUG proceedings. But for some firms, the German government has taken the position that it cannot let that happen. The German flagship carrier Lufthansa is an example. With the worldwide imposition of lockdowns and travel bans, it got into trouble in the first quarter of 2020. It was bailed out in May 2020 with a 9 billion euro rescue package from the German government, giving the latter a 20% equity stake in the struggling airline.^22^ Another example, now related to the ongoing energy crisis, is the nationalisation of Uniper, once Europe’s biggest importer of Russian gas.^23^ Uniper was brought to the brink of collapse after supply cuts by Russia, forcing it to buy more expensive gas on the spot market in order to meet its supply contracts. With a 29 billion euro rescue package, the German government bought out Uniper’s previous owner, recapitalised the entity and provided new cash.

The overall direction of the German policy response to the Covid-19 pandemic and the current energy crisis is clearly anti-bankruptcy: businesses receive enormous amounts of financial assistance to allow them to continue operating outside bankruptcy. Individual critical firms such as Lufthansa or Uniper are bailed out by the state and nationalised in whole or in part if the general assistance measures do not suffice to keep them afloat. The Insolvency Plan Procedure has been used during and after the pandemic as a restructuring tool only in relatively few cases, and the new StaRUG procedure has hardly been used at all. How should we assess this anti-bankruptcy policy of the German government?

In their contribution to this volume, Wolfram Prusko and David Ehmke argue that a policy focus should be on the reform of restructuring proceedings, and they make a number of suggestions in this regard: the system of mandatory filing duties should be reformed, the legal risks associated with rescue financing should be reduced, and the provisions supporting the filing and adoption of restructuring plans should be amended, amongst others.^24^ These are all sensible suggestions. It is always a good idea to improve bankruptcy (restructuring) proceedings, and I have suggested other reforms myself in this article, such as repealing the StaRUG and putting the focus on the Insolvency Plan Procedure. At the same time, in the context of shock events such as the Covid-19 pandemic or the current energy crisis, the anti-bankruptcy policy of the German government has merit in principle, as I will argue in the following.

### Structural Limits

First, take the millions of firms which have faced or are facing severe financial distress after the onset of the pandemic or during the current energy crisis. Both types of events severely affect the cash flow of businesses, but they do so differently. The pandemic led to lockdowns, and lockdowns caused businesses to experience a severe and abrupt *loss of revenue*.^25^ Businesses were (temporarily) prevented from trading. They could not make any (or only very little) money. The current energy crisis has created a different problem for businesses. The war in Ukraine has led to severe disruptions in supply chains and to rising energy prices for electricity and fossil fuels, in particular for gas. As a consequence, businesses face severe *cost increases*. Supplies have become (much) more costly, as has production, especially in energy-intensive industries. As demand for most goods and services is not completely inelastic, firms cannot recoup these cost increases in full by raising prices. A severe cash flow problem looms.

What can a bankruptcy restructuring do to solve these problems? Restructuring laws seek to modify the *financial structure* of a distressed business. Creditors’ claims are cut or exchanged for new (debt or equity) claims. The main goal usually is to reduce the debt of the company to a sustainable level. At the same time, in the context of a financial restructuring, often the business itself is restructured, i.e., an economic restructuring is undertaken. The goal of this is to increase the profit-generating capacity of the business or to help it return to profitability—as the case may be—by, for example, selling certain assets, closing down certain business lines, or developing new products. However, it is important to note that the legal technology which is central to a restructuring proceeding seeks to modify the financial claims against a business. It cannot change its economic structure.

Against this background, it becomes clear why a bankruptcy restructuring is not the right tool to help the millions of firms which are financially distressed because of geopolitical and/or macroeconomic shocks such as the Covid-19 pandemic or the current energy crisis. A bankruptcy restructuring cannot produce the missing revenues which firms have lost because of the lockdowns. The problem such a restructuring can solve is unsustainable debt levels. It cannot generate revenues. Similarly, a bankruptcy restructuring cannot force suppliers of goods and services to charge lower prices for their supplies. It cannot reduce energy costs either. Again, the problem which a bankruptcy restructuring can solve is unsustainable debt levels. It cannot change market prices for goods and services, in particular energy.

Hence, *structurally*, a bankruptcy restructuring is not the right tool to address the problems caused for businesses by the Covid-19 pandemic and/or the ongoing energy crisis. Other tools are needed, such as cash subsidies, loans or regulatory interventions in (energy) markets to limit price increases—and thus help the distressed businesses.

This is not to say that bankruptcy proceedings are unnecessary or should be avoided ‘at all costs’. Shock events such as the pandemic or the energy crisis have different effects on different firms. Businesses hit hardest are generally those which have the most vulnerable capital structure, i.e., the highest debt levels. Specifically with respect to the energy crisis, energy-intensive firms which massively use fossil fuels are at high risk. Some firms will not be able to avoid (imminent) insolvency despite the general financial assistance offered by the state, and these firms should enter a formal liquidation or restructuring proceeding. This implies that measures such as suspending filing duties or similar provisions, such as wrongful trading rules, are highly problematic. Distressed businesses should initiate formal bankruptcy proceedings if their continued trading poses a risk to existing and future creditors. So, in essence, bankruptcy is and should be the second but not the first line of defence against shock events such as the pandemic or the energy crisis.

Note that this argument is not an argument based primarily on the scale of the regulatory problem caused by these events. It is an argument based on the structural possibilities and limitations of bankruptcy (restructuring) laws. The influx of millions of distressed businesses into bankruptcy (restructuring) proceedings creates a different set of problems. First, even in restructurings, asset sales are usually part of the mix of adopted measures, and the simultaneous bankruptcy of millions of businesses would severely depress asset prices, leading to suboptimal results.^26^ Second, bankruptcy courts would be overwhelmed. In theory, this problem could be solved by providing much more resources to the bankruptcy system. In practice, this is not possible, at least not on short notice. And it would not be the best solution either. Bankruptcy (restructuring) proceedings involve significant curtailments of creditors’ claims. Procedures must contain due process safeguards, such as judicial oversight. If a restructuring is done on the basis of a structured bargaining procedure, the process takes some time and is usually procedurally intricate. Hence, bankruptcy (restructuring) proceedings come with certain fixed costs.

If one wanted to design a restructuring proceeding which is calibrated to manage the simultaneous influx of thousands or millions of similarly situated firms, it probably would be much simpler and less complex than the restructuring proceedings as we know them. Structured bargaining and class-wise voting as, for example, in Chapter 11 are too complex and costly. A simple majority rule based on debt value and a rule of equal treatment of all creditors—secured and unsecured—could be key features.^27^ Even such a procedure could not solve the fire-sale problem mentioned above, however.

### Critical Firms

The second manifestation of the anti-bankruptcy policy of the German government are the ad hoc bailouts of critical firms such as Lufthansa or Uniper, as described above. These bailouts are not mass events. To the contrary: it is single large firms which experience severe financial distress and which are rescued by the government. In principle, these cases appear to be perfect examples of the proper use of complex restructuring proceedings such as Chapter 11, the Insolvency Plan Procedure, or StaRUG. The size of the firms and their economic relevance certainly justify the costs of initiating and running a complex structured bargaining procedure. Nevertheless, the government decided to bail these firms out—for good reason?

The problems with using a bankruptcy (restructuring) procedure to manage the financial distress of these firms are again *structural*. Lufthansa was brought down by a severe case of revenue loss in 2020 as air travel came to an abrupt standstill during the first wave of the pandemic. Uniper suffered from exploding costs when the spot price for gas skyrocketed in the spring/summer of 2022. So, all the arguments discussed above to justify cash assistance by the government apply. But why were these firms deemed worthy of extraordinary help, going much beyond the assistance available to all firms? Why was a bankruptcy restructuring not even the second line of defence against the two shock events which have been and are disrupting lives and livelihoods worldwide?

Bankruptcy restructuring proceedings are structurally limited in what they can do. As discussed, these proceedings aim to rearrange the financial claims against the distressed entity. As a consequence, the key stakeholders in a corporate restructuring are the company’s creditors and the debtor corporation and its shareholders. The decision to continue running the firm as a going concern (in a restructured form) or to close it down is typically taken based on the effects of the decision on these financial stakeholders. The whole bankruptcy machinery of a ranking of claims, of voting, and of plan confirmation is calibrated such that the interests of those with a financial claim against the business are satisfied to the extent possible, under the circumstances, and according to the ordering of claims prescribed by the law.

What is typically not relevant is whether and to what extent the interests of those external to the bankruptcy process are affected by it.^28^ Bankruptcy is concerned with what can be described as ‘microeconomic efficiency’, i.e., firm-level efficiency, compared to a wider notion of efficiency which encompasses costs and benefits accruing to those who do not participate in the bankruptcy process and have no voice in it (‘macroeconomic efficiency’). Shutting down Lufthansa or Uniper, for example, may create significant negative effects on the wider economy and society which go much beyond the consequences for those who hold financial claims against these firms: business partners might be pulled into insolvency, regional or even national unemployment levels could rise, and the living conditions of many individuals be severely worsened. Conversely, maintaining these firms as going concerns could contribute to avoiding or at least containing these negative externalities. With respect to certain firms which perform important critical functions in and for a national economy, these effects can be of significantly greater magnitude than the effects of the firm’s distress on the firm level alone. But the bankruptcy process does not account for such positive or negative externalities. This is the structural reason the German government was justified in bailing out critical firms such as Lufthansa or Uniper. Bankruptcy is the wrong tool to restructure such firms. Their continued operation typically has significant positive, and their liquidation significant negative effects on the wider economy and society. Hence, ad hoc bailouts are a necessary crisis management tool.

Lufthansa and Uniper are non-financial firms. Many will recall that a similar policy discussion took place during and after the great financial and economic crisis regarding banks and other financial institutions. The key argument against using traditional bankruptcy procedures to deal with the financial failure of ‘systemically relevant’ financial firms was also based on externalities. It was feared that their collapse could bring the whole financial system down and cause havoc to the world economy.^29^ The emerging regulatory consensus at the time was that some kind of special restructuring regime for financial institutions was needed which would allow for a rapid recapitalisation. In Europe, this was achieved through the ‘Bank Recovery and Resolution Directive’.^30^ This points to the direction the discussion on bailouts of non-financial firms should take: we need to think about the legal framework which governs such bailouts.

## Improving Bailout Frameworks

Corporate bankruptcy proceedings are heavily regulated, and they must be, given that constitutionally protected rights—the financial claims of creditors and shareholders on the assets of the insolvent corporation—are at stake. In restructuring proceedings, detailed provisions seek to make sure that priority rules are observed and that all creditors and the debtor are treated fairly—however that is defined in a particular jurisdiction. The case law in leading restructuring jurisdictions such as the United States or England regarding specific aspects of the applicable legal regime is rich and dense. Structured bargaining procedures such as Chapter 11 in the US are a good example of this. Chapter 11 is a heavily litigated part of federal law. Nuances of intricate legal concepts have been fleshed out by the courts over decades.

The contrast to ad hoc bailouts of critical firms, such as Lufthansa or Uniper discussed above, could not be starker. These bailouts are essentially business deals. The core commercial terms (who gets/pays what?) are subject to ad hoc negotiations instead of being meticulously regulated by statute. For sure, these bailouts do not take place in a legal vacuum. There are legal constraints which need to be observed, stemming mostly from corporate and competition law. But there are no rules which determine the payoffs of the financial stakeholders of the distressed enterprise. Regarding Uniper, for example, the German government decided, in July 2022, to take a 30% stake in the energy firm, make up to 7.7 billion euro available as hybrid capital and expand a credit line to 9 billion euro through the state-run bank KfW.^31^ Two months later, the government announced that it would completely take over Uniper, buying out the current Finnish owner Fortum for approximately 0.5 billion euro.^32^ Currently, the accountability for the use of taxpayer funds in this as in other such ad hoc bailouts is almost entirely political and not legal.

This is all the more problematic as ad hoc bailouts of this magnitude raise important and difficult economic and justice questions. Economically, there is the well-known ‘too big to fail’ problem and the moral hazard created *ex ante* if a bailout in case of a serious crisis is a likely prospect.^33^ There are also potentially significant competitive distortions caused by massive subsidies. The applicable rules on state aid are often riddled with exceptions in times of severe crisis or suspended altogether, providing little protection against such distortions.^34^

However, the most crucial issues are fairness and justice questions. These relate to the distribution of losses and profits amongst the (financial) stakeholders of a distressed critical firm and the general taxpayer. Lufthansa and Uniper used to be highly profitable enterprises, generating large profits for its shareholders. What is the justification for paying Fortum 0.5 billion euro in the Uniper bailout and for injecting further billions of euros of taxpayer money into the firm, bailing out the firms’ existing creditors? Are profits privatised and losses socialised in this and in similar cases? What is the distributive metric which should be applied to achieve a fair distribution of gains and losses? What rules should be in place if the entity in question returns to profitability?^35^

If and to the extent that ad hoc bailouts of critical firms are becoming more frequent, these questions demand to be answered. At the very least, attempts must be made to reduce the discrepancy between heavily regulated restructurings in bankruptcy on the one hand and almost completely unregulated ad hoc bailouts on the other hand. If these discrepancies persist, it is to be expected that, on the margin, firms will increasingly attempt to ‘game the system’, for example, by incurring wasteful expenditure—empire building, lobbying the media, threatening mass layoffs, deindustrialisation of regions, etc.—to become eligible for an ad hoc bailout in case they find themselves in serious financial distress.

It is likely that geopolitical and macroeconomic shocks such as the Covid-19 pandemic or the current energy crisis will indeed become more frequent in the years to come. Climate change threatens lives and livelihoods, and might well trigger mass migration in the near future. Democracy is under threat from authoritarian leaders around the world. Wars could become more frequent. Maintaining interest rates at extraordinarily low levels for years has contributed to an extraordinary rise in asset prices and a significant increase in (public and private) debt. The current energy crisis has exacerbated the problem. Falling asset prices might initiate the next global financial and economic crisis.^36^

Improving the bailout framework for critical firms could start with work by institutions which, for a long time, have been active in developing Conventions, Model Laws, Legislative Guides and ‘Principles’ which seek to modernise and harmonise rules in the area of (transnational) commercial law. Chief among these institutions are UNIDROIT in Rome, UNCITRAL in Vienna/New York, and ELI in Vienna. UNCITRAL has developed a ‘Model Law on Cross-Border Insolvency’ (1997), a ‘Legislative Guide on Insolvency Law’ (starting in 2004), and a ‘Model Law on Enterprise Group Insolvency’ (2019), for example.^37^ UNIDROIT has developed ‘Principles of International Commercial Contracts’ (2016) and ‘Principles of Transnational Civil Procedure’ (2006), for example.^38^ ELI has produced Model Rules for ‘Rescue of Business in Insolvency Law’ (2017).^39^

These institutions could start work on ‘Principles on Ad Hoc Bailouts of Critical Firms’.^40^ The idea would be to draw on existing legislative sources—such as, for example, rules on state aid, restrictions on foreign direct investment or on the Golden Shares’ jurisprudence of the CJEU^41^—to distil underlying principles which might determine which firms should be candidates for such bailouts, and on what conditions. The comparative analysis of these sources could be further informed by case studies on large bailouts of critical firms and lessons learned from them. If and to the extent that the principles draw from different legal sources and case studies from different jurisdictions and legal spheres, they could of course not claim to provide precise legal guidance on how such bailouts should be conducted. But that would not be their purpose. Their purpose would be to provide guideposts for regulatory action and policy discourse and to make a first step from the status quo of a strikingly underregulated field towards a new regime of some form of legal and not just political accountability for ad hoc bailouts of critical firms.

Another avenue for reform could seek to strengthen firms’ resilience against macroeconomic or geopolitical shocks such as the Covid-19 pandemic or the current energy crisis. This avenue would aim to help businesses weather a storm once it occurs by increasing their shock-absorbing capacity *ex ante* instead of improving the applicable legal framework *ex post*, i.e., in a time of crisis. The main goal in this context would be to improve the equity capital position of firms, i.e., to reduce their leverage such that shocks/losses could be absorbed better.

Inspiration in this regard can be drawn from the Basel process, in particular from the Basel Committee on Banking Supervision which has been active for decades ‘to enhance financial stability by improving the quality of banking supervision worldwide’.^42^ The Basel III international regulatory framework for banks^43^ has been adopted in the EU, for example, with the Capital Requirements Regulation and Directive in 2013.^44^ It seeks to strengthen the resilience of banks by improving their regulation, supervision and risk management. A key element of the framework is minimum capital requirements.^45^

In the context of non-financial firms, the debate about minimum capital requirements for corporations also has a long history.^46^ This is true especially in the EU where such requirements (and distribution restrictions) are still a key plank of the applicable regulatory framework for large corporations: public limited liability companies must have a minimum capital of 25,000 euro, and they are subject to meaningful distribution restrictions.^47^ These requirements could be strengthened significantly. I am certainly not claiming that stronger minimum capital requirements and distribution restrictions could or would make ad hoc bailouts of critical firms superfluous. But they could be an element of a more comprehensive regulatory mix to improve the resilience of these firms.

Another element could be the establishment of a bailout fund which would be financed by regular contributions from businesses. The key idea behind this proposal is twofold: to increase the involvement of the private sector in the financing of bailouts, partially correcting the current situation which, arguably, is characterised by a privatisation of gains and a socialisation of losses; a second benefit of such a fund could be to delay or even make superfluous (in some instances) state intervention and help, if and to the extent the fund is able to reduce or even eliminate the financial distress of affected businesses. In essence, the fund would provide a form of insurance against financial distress for its contributing members. Similar schemes exist in many jurisdictions already, albeit with a relatively small scope. In Germany, for example, the *Pensions-Sicherungs-Verein* has existed since 1975.^48^ It receives contributions from member firms (currently more than 100,000). Its goal is to make sure that pension schemes operating at the level of an individual business are not affected by an insolvency of that business. This idea could be expanded to cover the financial consequences of distress situations caused by macroeconomic or geopolitical shock events more broadly, at least partially.

Finally, a much more ambitious (and controversial) move could be to restrict the uses to which private corporations can be put. Bailing out firms with taxpayer money only becomes an issue for firms which are run as private corporations in the first place. In August 2018, Senator Elizabeth Warren introduced the ‘Accountable Capitalism Act’ to the 115th US Congress.^49^ Under the proposed Act, very large American corporations (more than 1 billion US dollars in annual revenue) would have to obtain a federal charter as a ‘United States corporation’, which obliges company directors to consider the interests of all corporate stakeholders, such as employees.

One can imagine extending this idea to exclude certain business activities from being conducted in the form of a private corporation in the first place. If ‘critical infrastructure’ firms were only run as public entities, the discrepancy between a privatisation of losses and a socialisation of gains would not arise. And controlling these entities for the benefit of the whole population, especially in crisis situations, would be much easier. Of course there would be other issues. Such firms would then not be subject to the discipline of the market’s competitive pressures, possibly lowering the efficiency with which they are run. And a multiplicity of private actors might bring more diversity and risk diversification than a state-run monopolist. Societies will need to carefully balance these effects when making design choices regarding the governance structure of such enterprises. On the margin, given the increased frequency of geopolitical and/or macroeconomic shocks, running critical firms as state-owned enterprises should become more attractive.

## Conclusion

Geopolitical and macroeconomic shocks such as the Covid-19 pandemic and the war in Ukraine and, as a consequence, the current energy crisis are threatening lives and livelihoods across the globe. More such shocks are to be expected in the years to come, especially in the context of the global climate crisis. Businesses are struggling, suffering from dramatic losses of revenue and/or cost increases. Governments around the world attempt to contain the financial damage by offering massive amounts of financial aid in a dramatic attempt to keep firms operating and out of bankruptcy. If the problems become too severe, critical firms are often bailed out by the state as, for example, Lufthansa or Uniper in Germany. The worldwide policy trend appears to be clearly anti-bankruptcy. Is this trend justified?

In this article, I have looked at this question from the perspective of Germany, as do Wolfram Prusko and David Ehmke in their contribution to this volume. I have argued that the new German restructuring law, the StaRUG, is a superfluous and flawed instrument. It should be repealed. I have also argued that bankruptcy laws, including restructuring laws, are generally ill-suited to deal with macroeconomic shock events. At least they cannot and should not provide the ‘first line of defence’. Bankruptcy laws are not designed to provide the structural assistance at scale which the businesses affected by these shock events need. Hence, the post-Covid anti-bankruptcy policies of many states, including Germany, are justified in principle. At the same time, ad hoc bailouts are also problematic. I have proposed various measures to increase firms’ resilience against macroeconomic shock events, and I have suggested that best practices (‘Principles’) for such bailouts should be developed.

What we are currently observing with respect to corporate insolvency and restructuring law may just be a manifestation of a much bigger problem. It is not just the case that the tools of corporate insolvency and restructuring law come to their limits when the challenge is to manage the economic and financial consequences of geopolitical and macroeconomic shock events. Rather, a wider crisis of the economic institutions of capitalism could be in the making. We need to fundamentally rethink the delineation of private and public activities. Should critical firms be run as private entities? Should we restrict access to the corporate form more generally? How should private corporations be governed? What role should the state/private sector play when geopolitical turmoil—weather catastrophes, wars, mass migration, etc.—becomes an almost daily occurrence? Is it time for a (new) socialism?^50^ These are tough questions, and there are no easy answers. What seems clear, though, is that the state is back on the stage as a powerful economic actor.^51^ The night-watchman state is clearly a thing of the past.

## Footnotes

1 For a historical and analytical account of reorganisation practice under Chapter 11, see Skeel (2001), Chapters 6 and 8, Lubben (2020), and Baird (2022).

2 Directive (EU) 2019/1023 of the European Parliament and of the Council of 20 June 2019 on preventive restructuring frameworks [2019] OJ L 172/18-55. On the European ‘restructuring policy‘ from a US perspective, see Parikh (2020).

3 Act on the Stabilisation and Restructuring Framework for Businesses (Unternehmensstabilisierungs- und -restrukturierungsgesetz – StaRUG) of 22 December 2020, BGBl. I, p 3256.

4 Prusko and Ehmke (2023).

5 See Eidenmüller (2019), Annotations before §§ 217-269 InsO, margin no. 19.

6 See Federal Statistical Office, Insolvenzen von Unternehmen und Übrigen Schuldnern, https://www.destatis.de/DE/Themen/Branchen-Unternehmen/Unternehmen/Gewerbemeldungen-Insolvenzen/Tabellen/anzahl-der-beantragten-insolvenzverfahren.html;jsessionid=C17C7C0E52749049C2E3865197B98F3C.live712 (last visited on 11 November 2022).

7 See Institut für Mittelstandsforschung, Eigenverwaltungen in Unternehmensinsolvenzverfahren, https://www.ifm-bonn.org/fileadmin/data/redaktion/statistik/gruendungen-und-unternehmensschliessungen/dokumente/Eigenverwaltung_D_1999-2021.pdf (last visited on 11 November 2022).

8 See Börsennews, Sanierung: Insolvenzverfahren der Sunline AG beendet, https://www.boersennews.de/community/blog/sanierung-insolvenzverfahren-der-sunline-ag-beendet/5739/ (last visited on 11 November 2022).

9 See Paulus et al. (2010).

10 Ibid.

11 It is telling that Clemens Fuest, who heads the ifo Institute, a leading economic policy advisory institution (see https://www.ifo.de/en (last visited on 28 November 2022)), does not even discuss the (de)merits of bankruptcy proceedings in his book on the appropriate economic policy response to the pandemic. See Fuest (2020).

12 See INDat Report, ‘INDat Report ermittelt Zwischenbilanz zu einem Jahr Restrukturierungsrahmen’, Pressemitteilungen, 1 February 2022, https://www.indat.info/Meldungen/Pressemitteilungen-Insolvenz-Sanierung/INDat-Report-ermittelt-Zwischenbilanz-zu-einem-Jahr-Restrukturierungsrahmen-9a7524621d24d41685599de7ef01418a#:~:text=Die%20R%C3%BCckmeldungen%20aller%2024%20Gerichte,in%20einem%20gerichtlich%20best%C3%A4tigten%20Restrukturierungsplan (last visited on 12 November 2022).

13 On 10 October 2022, Professor Stephan Madaus estimated the total number of StaRUG proceedings since 1 January 2021 to be approximately 35 (email to author dated 10 October 2022). The (precise) number for 2021 is 22, see previous footnote. Hence, the number for 2022 until mid-October, based on Professor Madaus’s estimate, is 13.

14 See, for example, Amtsgericht Köln, Resolution dated 3 March 2021, Az. 83 RES 1/21, https://rewis.io/urteile/urteil/via-03-03-2021-83-res-121/ (last visited on 13 November 2022).

15 German insolvency administrators form a powerful lobby group, the ‘Verband Insolvenzverwalter und Sachwalter Deutschlands’, https://www.vid.de/ (last visited on 13 November 2022).

16 The key lobby group of these professionals is the ‘Gesellschaft für Restrukturierung – TMA Deutschland’, https://www.tma-deutschland.org/organisation-8.html (last visited on 13 November 2022).

17 See, for example, Jörn Poppelbaum, Den Bock zum Gärtner machen, 22 July 2003, https://www.juve.de/verfahren/insolvenzticker-den-bock-zum-gaertner-machen/ (last visited on 13 November 2022).

18 See Reuters, ‘Lehman Germany’s administrator to earn record fee’, 22 November 2012, https://www.reuters.com/article/lehman-germany-idUSL5E8MMDVF20121122 (last visited on 13 November 2022).

19 See TMA Press Release, ‘TMA fordert schnelle Umsetzung des StaRUG’, 10 November 2020, https://www.tma-deutschland.org/presse-nf-leser/tma-fordert-schnelle-umsetzung-des-starug.html (last visited on 28 November 2022).

20 See Niering, Stellungnahme von Dr. Christoph Niering, Vorsitzender des Verbandes Insolvenzverwalter Deutschlands e.V. (VID), 23 November 2020, https://www.vid.de/wp-content/uploads/2020/11/Stellungnahme-v.-Dr.-Christoph-Niering-VID-zur-Sachverstaendigenanhoerung-zum-RegE-des-SanInsFoG.pdf (last visited on 28 November 2022).

21 Reuters, ‘Germany’s parliament approves 200 billion euro fund to tackle energy crisis’, 21 October 2022, https://www.reuters.com/business/energy/germanys-parliament-approves-200-billion-euro-fund-tackle-energy-crisis-2022-10-21/ (last visited on 13 November 2022).

22 Joe Miller, ‘German government agrees €9bn bailout for Lufthansa’, Financial Times, 25 May 2020, https://www.ft.com/content/3e227f73-d421-4b9e-bd57-1723d16becdb (last visited on 13 November 2022).

23 Olaf Storbeck et al., ‘Germany nationalises struggling utility Uniper in €29bn bailout’, Financial Times, 21 September 2022, https://www.ft.com/content/2190babb-1cb8-49f1-a9d5-2c9d98539de9 (last visited on 14 November 2022).

24 Prusko and Ehmke (2023), Sections IV 1–3.

25 See van Zwieten et al. (2021), pp 200–201.

26 Shleifer and Vishny (2011).

27 The English ‘Company Voluntary Arrangement’ features such a majority rule (75% of debt value), but it does not apply to secured creditors.

28 See Eidenmüller and Paz Valbuena (2021), pp 521–523.

29 See Eidenmüller (2018), pp 1008–1010.

30 Directive (EU) 2014/59 of the European Parliament and of the Council of 15 May 2014 establishing a framework for the recovery and resolution of credit institutions and investment firms [2014] OJ L 173/190-348.

31 See Deutsche Welle, ‘Germany finalizes bailout of energy firm Uniper’, 22 July 2022, https://www.dw.com/en/germany-finalizes-bailout-of-ailing-energy-giant-uniper/a-62562327 (last visited on 18 November 2022).

32 See Fortum, ‘Fortum to fully divest Uniper to the German state’, 21 September 2022, https://www.fortum.com/media/2022/09/fortum-fully-divest-uniper-german-state#:~:text=In%20connection%20with%20the%20capital,98.5%25%20of%20Uniper (last visited on 18 November 2022).

33 See generally Sorkin (2010).

34 See, for example, the ‘Temporary Crisis Framework’ adopted by the European Commission on 23 March 2022 to support the economy in context of Russia’s invasion of Ukraine, https://ec.europa.eu/commission/presscorner/detail/en/statement_22_1949 (last visited on 18 November 2022).

35 The German government, for example, sold its 20% stake in Lufthansa, acquired during the pandemic, in September 2022. See Reuters, ‘German government sells remaining Lufthansa shares’, 13 September 2022, https://www.reuters.com/business/aerospace-defense/german-government-intends-reduce-lufthansa-stake-further-bank-2022-09-13/ (last visited on 15 December 2022). Was this good timing? Could a (much) higher price have been achieved by holding the stock longer? Lufthansa’s share price rose by over 25% from 13 September to 13 December 2022, see https://investor-relations.lufthansagroup.com/en/share-bonds/share-price.html (last visited on 15 December 2022).

36 King (2017), Ch. 9.

37 See United Nations Commission on International Trade, Insolvency, https://uncitral.un.org/en/texts/insolvency (last visited on 20 November 2022).

38 See UNIDROIT Principles 2016, https://www.unidroit.org/instruments/commercial-contracts/unidroit-principles-2016/ (last visited on 20 November 2022).

39 See European Law Institute, Rescue of Business in Insolvency Law (6 September 2017), https://www.europeanlawinstitute.eu/about-eli/bodies/membership/default-title/rescue-of-business-and-insolvency-law/ (last visited on 21 November 2022).

40 See Eidenmüller and Paz Valbuena (2021), pp 523–535.

41 Ibid., pp 514-520, with further references.

42 BIS, History of the Basel Committee, https://www.bis.org/bcbs/history.htm?m=3076 (last visited on 21 November 2022).

43 BIS, Basel III: international legal framework for banks, https://www.bis.org/bcbs/basel3.htm (last visited on 21 November 2022).

44 Regulation (EU) 575/2013 of the European Parliament and of the Council of 26 June 2013 on prudential requirements for credit institutions and investment firms [2013] OJ L 176/1-337. Directive (EU) 2013/36 of the European Parliament and of the Council of 26 June 2013 on access to the activity of credit institutions and the prudential supervision of credit institutions and investment firms [2013] OJ L 176/338-436.

45 BIS, Minimum capital requirements for market risk, https://www.bis.org/bcbs/publ/d457.htm (last visited on 21 November 2022).

46 See the contributions in Eidenmüller and Schön (2008).

47 See Articles 45 and 56 of Directive (EU) 2017/1132 of the European Parliament and of the Council of 14 June 2017 relating to certain aspects of company law [2017] OJ L 169/46-127.

48 See PSVaG, https://www.psvag.de/wir-ueber-uns/ziel-und-zweck.html (last visited on 21 November 2022).

49 See CONGRESS.GOV, S.3348 – Accountable Capitalism Act, 115th Congress (2017-2018), https://www.congress.gov/bill/115th-congress/senate-bill/3348/text (last visited on 21 November 2022).

50 Piketty (2021).

51 See Mazzucato (2021).
